# Robot assisted radical cystectomy versus open radical cystectomy in bladder cancer (RACE): study protocol of a non-randomized comparative effectiveness study

**DOI:** 10.1186/s12885-018-4779-6

**Published:** 2018-09-03

**Authors:** C. J. Wijburg, C. T. J. Michels, J. R. Oddens, J. P. C. Grutters, J. A. Witjes, M. M. Rovers

**Affiliations:** 1 Department of Urology, Rijnstate Hospital, Wagnerlaan 55, 6815 AD Arnhem, The Netherlands; 2 Department of Urology, Amsterdam UMC, University of Amsterdam, Meibergdreef 9, 1105 AZ Amsterdam, The Netherlands; 3 Departments of Operating Rooms and Health Evidence, Radboud university medical center, Geert Grooteplein Noord 21, 6500 HB Nijmegen, The Netherlands; 4 Department of Urology, Radboud university medical center, Geert Grooteplein Noord 21, 6500 HB Nijmegen, The Netherlands

**Keywords:** Cost-effectiveness, Robot-assisted, Radical cystectomy, Bladder cancer, Complications, Quality of life

## Abstract

**Background:**

Despite the fact that the cost-effectiveness of robot-assisted radical cystectomy (RARC) is not yet proven, and open radical (ORC) cystectomy is recommended as the standard of care in patients with high-risk non-muscle-invasive and muscle-invasive bladder cancer, the use of RARC is still increasing. The objective of the current ongoing comparative effectiveness trial therefore is to study the (cost-)effectiveness of RARC compared to ORC, both in terms of objective (complication rates, oncological outcomes) and patient-reported (health-related quality of life) outcome measures.

**Methods:**

This study is designed as a non-randomized, multicentre comparative effectiveness trial.

Centres with an annual caseload of > 20 radical cystectomies can include patients after informed consent has been given. Centres that perform RARC must have passed the (initial) learning curve of 40 cases. A total of 338 (2 × 169) patients will be enrolled from 23 participating centres (12 ORC, 10 RARC and 1 LRC). Follow-up visits will be scheduled at 1, 3, 6 and 12 months. During each follow-up visit, clinical data and health-related quality of life questionnaires will be administered. Costs will be studied using a monthly resource usage questionnaire. Impact on complications and quality of life will be calculated as the average difference between the groups with 95% confidence intervals, adjusted for potential baseline differences by means of propensity score matching.

**Discussion:**

This study aims to contribute to the development of evidence-based guidelines regarding the most cost-effective surgical technique for radical cystectomy.

**Trial registration:**

Nederlands Trial Register/Dutch Trial Registry, trial identifying number: NTR5362. Registered on 14 August 2015. (http://www.trialregister.nl/trialreg/admin/rctview.asp?TC=5362).

## Background

Radical cystectomy with pelvic lymphadenectomy and urinary diversion is the standard of care for both high-risk non-muscle-invasive bladder cancer and muscle-invasive disease [1]. This surgery is complex and associated with considerable postoperative morbidity, including major and minor complications with probabilities ranging from 13 to 67% [2–6]. Complications may lead to extra costs, prolonged hospital stay, additional interventions, a reduced quality of life, and a longer time before patients may return to normal activities.

Currently, there are two main surgical techniques to perform a radical cystectomy: robot-assisted (RARC) and open surgery (ORC). ORC is the recommended treatment according to European guidelines, but RARC has been suggested to improve the perioperative morbidity without compromising oncological efficacy, and the percentage of hospitals and surgeons performing RARC is increasing steadily [7]. For example, 11 out of 69 (14.4%) Dutch centres performed RARC in 2014, and in 2016 24% of all radical cystectomies were performed robot assisted [8]. In the USA, the use of robot assisted technique has increased from 1% in 2004 to 13% in 2010 [9]. High-level evidence of methodologically sound clinical studies that compare both treatments is, however, lacking. Furthermore, direct costs of robotic surgery are expected to be higher than for open surgery, so investment in this technology can only be justified when additional costs are balanced by a significant benefit regarding patient outcomes.

### Existing knowledge

At the time we designed this study (2015), two systematic reviews (both including 105 studies) had been reported. [2, 10] Novara et al. reported on perioperative outcomes and complications after RARC, whereas Yuh et al. reported on oncologic and functional outcomes after RARC. Ninety-three of the 105 studies reported perioperative outcomes and complications of RARC; including 23 comparative studies and 70 surgical series. Most of the surgical series were retrospective single centre studies and only three randomized trials were found.

The overall results showed shorter operative time for ORC, whereas blood loss and hospital stay were better with RARC. Moreover, 90 day complication rates of any grade and grade 3 complications were lower for RARC, whereas high-grade complication and mortality rates were similar [2] Analyses showed no significant difference between RARC and ORC in lymph node yield and rates of positive surgical margins [10]. Very limited data were available with respect to functional outcomes and quality of life. According to both authors, the most relevant limitations of their systematic reviews were the quality of the included studies, the small study populations, the retrospective nature of most series, the short follow-up period, and the lack of standardized definitions [2, 10]. This lack of methodologically sound data precludes a definite conclusion about the effectiveness of RARC versus ORC.

Up to now, four randomised trials [11–14] have been reported and two others are ongoing (RAZOR [15] and iROC (ClinicalTrials.gov identifier: NCT03049410). Shen et al. [16] performed a meta-analysis of these 4 RCT’s [11–14] and presented evidence for a benefit of estimated blood loss, time to diet, similar perioperative complications and oncological outcomes, but a longer operative time in RARC. Furthermore, in the largest trial [13] surgeons performing RARC were still in their learning curve, and another trial only included 20 patients per arm and potential surgeon bias (i.e. there was a different surgeon for each technique) could not be precluded [14].

The two ongoing trials, i.e. RAZOR [15] and iROC, comprise multi center trials. RAZOR is a multi-institutional, randomized, non-inferiority, phase III trial, which aims to enrol 320 patients from 15 participating centres in the USA. They will study oncological outcomes, complications and quality of life of RARC compared to ORC. The primary endpoint is progression-free survival after 2 years. A potential limitation of the RAZOR trial might be the RARC learning curve, as surgeons must have performed only more than 10 RARCs in the past year. The learning curve of RARC is, however, estimated to be between 16 and 30 cases [17–19]. iROC is a prospective multicentre randomised controlled trial comparing the outcomes of intracorporeal RARC (iRARC) with ORC, which aims to enroll 320 patients. The primary outcomes (functional recovery and return to normal activities) will be measured 90 days post-surgery.

### Need for a comparative-effectiveness trial

As described above, all trials performed so far have been limited by sample size and design, or by their application of RARC with extra-corporeal reconstruction. We have chosen for a comparative effectiveness study since observed effectiveness will depend on the complex decision-making process of clinicians selecting therapies, their experience with the technique under study, and on the decisions and actions of patients, including whether they accept or prefer a certain surgical technique. The impact of these issues on effectiveness may be difficult or impractical to assess in a traditional RCT, in which treatment assignments are randomized and may therefore lead to a bias due to the expertise of the surgeons in a center.

### Objective

The objective of the current ongoing comparative-effectiveness trial is to study the effectiveness of RARC as compared to ORC in patients with bladder cancer and an indication for radical cystectomy in terms of complication rates, health related quality of life, and disease free survival.

## Methods

### Design

This study is designed as a non-randomized, multicentre comparative effectiveness trial. Patient recruitment is currently being conducted at urology outpatient clinics in five academic tertiary referral centres and eighteen referral centres in the Netherlands. A multicentre approach was chosen to obtain the desired power and to assure representativeness of our sample to the target population. Each centre will perform either RARC or ORC. Centres that perform both RARC and ORC can only participate if they do not select a technique based on patient characteristics, other than described in the exclusion criteria. Only high volume centres (> 20 radical cystectomies per year) can participate in the study. To prevent learning curve bias, only centres that have performed at least 40 RARCs can participate.

### Characteristics of participants

#### Inclusion criteria

Patients eligible for the trial must comply with all of the following:

1. Eighteen years or older.
2. Oncological indication for radical cystectomy.
3. Histologically proven primary muscle invasive transitional cell carcinoma or therapy resistant high-risk non muscle-invasive bladder cancer (CIS, refractory pTa-1).
4. Tumour is considered non metastatic (cT1a-cT4a, N0 M0) at the time of inclusion. Patients with N+ status may receive neoadjuvant chemotherapy, but can only be included in the study when preoperative scans show N0.
5. Able to fill in Dutch questionnaires.
6. Provide written informed consent.

#### Exclusion criteria

When a patient meets one of the following exclusion criteria, they will be excluded from participation:

1. Previous major abdominal surgery (i.e. existing stomata, status after low anterior resection of the rectum or rectal amputation, status after open aortabifemoral graft, status after right hemicolectomy).
2. Morbid obesity (BMI ≥ 40 kg/m2).
3. Radical cystectomy is performed in combination with a nephrectomy or a partial colon resection.
4. Pregnancy.

### Interventions

Overall, the surgical techniques for ORC or RARC are standardized procedures. Radical cystectomy with pelvic lymphadenectomy with diversion will be performed in all cases. If the formation of a neobladder is oncologically safe and technically feasible, patients can choose between an ileal conduit and a continent urinary diversion (neobladder). For RARC, one centre performs an extracorporeal technique, whereas all other centres perform a total intracorporeal reconstruction.

The peri-operative protocol has been standardized for all participating centres, including a fast track (ERAS) protocol. All centres are asked to follow the RACE-ERAS protocol, based on the current international guidelines and best available evidence. An identical protocol is used for RARC and ORC, to prevent bias due to different perioperative protocols. Table 1 presents the RACE-ERAS protocol used for this study.

**Table 1:** ERAS protocol used for RACE study

Item	Standard	Optional
1. Pre-operative: extensive counselling patient	✓	
2. Correction anaemia (Hb ≥ 6 mmol/l)	✓	
3. Pre-operative consult dietary specialist		✓
4. Quit smoking		✓
5. Physical exercise before surgery		✓
6. Pre-operative enema: 12 h before surgery (in case of constipation)		✓
7. Pre-operative: High calorie fluids, until 2 h before surgery	✓	
8. Pre-operative: solid food until 6 h before surgery	✓	
9. Pre-operative: clear fluids, until 2 h before surgery	✓	
10. Epidural analgesia (thoracal) until max 72 h after surgery	✓	
11. Antibiotic prophylaxis, one-shot pre-operative Cefazoline (≤80 kg 1 g; > 80 kg 2 g) + Metronidazole (500 mg). May be repeated if the operation time is longer than the half-life	✓	
12. Anaesthetic considerations Normothermia, Goal directed fluid therapy, normovolemia, short acting sedatives, minimalize opioids.	✓	
13. Post-operative: nasogastric tube removed directly after surgery	✓	
14. Post-operative: start laxantia post-op day 1	✓	
15. Post-operative: chewing gum		✓
16. Post-operative: standard anti-emetica	✓	
17. Post-operative: start mobilisation 6 h after surgery	✓	
18. Post-operative: fluid diet until first stool	✓	
19. Post-operative: Thrombosis prophylaxis for four weeks with ‘low molecular weight heparin’ = LMWH (Fraxiparine 0,6 cc)	✓	

### Outcomes

#### Primary endpoint

The primary outcome measure comprises 90 days complications, according to the Clavien-Dindo classification [20].

#### Secondary endpoints

Secondary outcomes comprise perioperative morbidity and mortality, 30 days and 1 year complications, health-related quality of life, disease free survival, time to return to normal activity, blood loss and transfusion parameters, operating time, hospital and intensive care stay, oncological outcomes, and costs.

#### Patient reported outcomes

Health-related quality of life is measured at baseline (pre-operatively) and post-operatively at 1, 3, 6, and 12 months using three quality of life questionnaires: Functional Assessment of Cancer Therapy–Bladder cancer Cystectomy (FACT-Bl-Cys, formerly FACT-VCI), Bladder Cancer Index, and the 5-level version of the EuroQol 5D (EQ-5D-5 L).

The FACT-Bl-Cys questionnaire is a disease and treatment-specific tool for assessing quality of life following radical cystectomy and urinary diversion [21]. It includes 42 questions dealing with general domains (physical, social/family, emotional and functional well-being) plus 17 additional items covering urinary, sexual, and bowel function. The Bladder Cancer Index is developed for patients who are diagnosed with bladder cancer and covers a broad range of health and quality of life, including three domains, namely urinary, bowel and sexual functioning, containing 14, 10 and 12 items, respectively [22]. The EQ-5D-5 L consists of a descriptive part and a visual analogue scale (VAS) [23]. The descriptive part comprises the following five dimensions: mobility, self-care, usual activities, pain/discomfort, and anxiety/depression. The VAS records the respondent’s self-rated health on a vertical scale ranging from 0 (worst imaginable health state) to 100 (best imaginable health state).

Resource use will be assessed using the RACE healthcare usage questionnaire, which is based on the iMCQ [24] (to assess medical consumption such as hospital visits, medication use and domestic help) and the iPCQ (to measure absence from work due to illness) [25]. This questionnaire will be completed at baseline (pre-operatively) and every month afterwards. It records resource use, such as doctor’s visits, medication, hospital admissions, and out-of-pocket expenses such as over-the-counter drugs. Additionally, travelling time to the outpatient clinic and related costs are asked for. Where relevant, (missing) entries will be verified by data from the medical records.

#### Clinical outcomes

Perioperative and postoperative measures (e.g. blood transfusion rates, intraoperative fluid requirements, operative time, length of hospital stay and analgesic requirement) are prospectively recorded. Operating time is defined as the skin-to-skin operating time in minutes, not including anaesthetic preparations as these might differ between the participating centres. Total operating room occupation time is also measured.

Pathological data is obtained from pathology reports after surgery with particular emphasis on surgical margin status, total number of lymph nodes removed and their involvement with cancer, as well as pathological stage of the tumour. A standardized form will be used to collect all information pertaining to specimen processing and staging by the participating institutions. In addition, since centres are obliged to complete the national urologic database, the Dutch Urological Association can and will provide us with export files for each RACE participant, which will subsequently be imported in the eCRF (electronic Case Report Form).

### Participant timeline

**Fig. 1:**
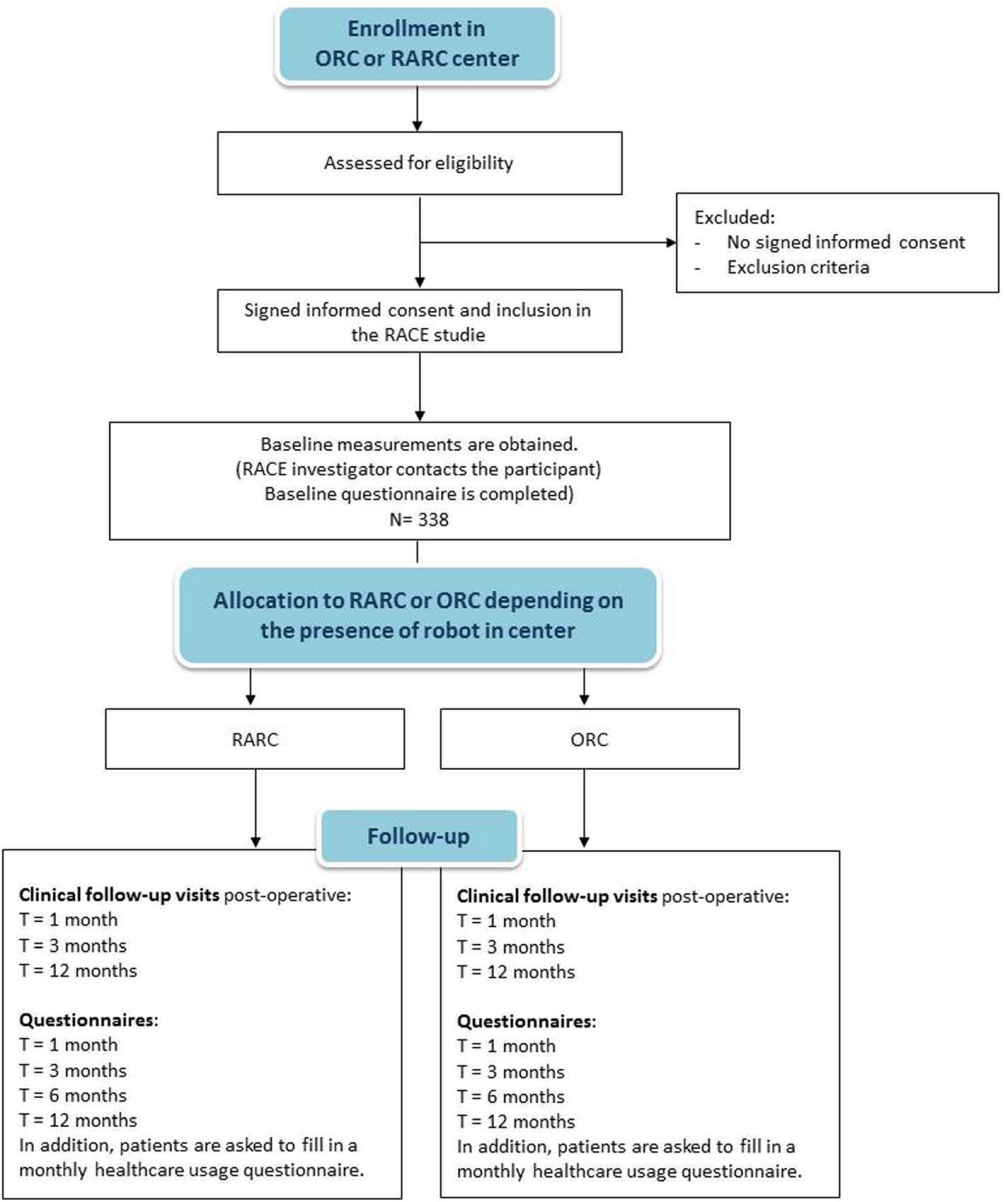
Flowchart RACE study

Timeline is depicted in Fig. 1. Treating urologists in the participating centres will recruit patients that fulfil the in- and exclusion criteria. Information is given about the rationale and the design of the RACE study. Potential participants will receive both the information letter and the informed consent form. Patients will have sufficient time to consider participation. If they agree to participate, informed consent will be signed. Once patients signed informed consent, contact information will be transmitted to the coordinating researcher. Patients will be operated in their own centre within 4 to 6 weeks; from the moment they signed informed consent.

Patients are asked to fill in 3 quality of life questionnaires at five different time points: before the operation, and then 1, 3, 6 and 12 months after surgery, which will take about ten minutes per questionnaire. In addition, patients are asked to fill in a cost questionnaire monthly, which will take approximately less than five minutes.

Clinical data are registered in a central validated database (eCRF, Research Manager) by (local) researchers. Clinical consultations are scheduled 1, 3, and 12 months after surgery. The data acquisition is presented in Table 2.

**Table 2:** Standard Protocol Items: Recommendations for Interventional Trials (SPIRIT) flow diagram of study enrolment, interventions and assessments

	Study period					
	Enrolment	Allocation	Post-allocation			Close-out
Timeline	4–6 weeks before Radical Cystectomy	Radical Cystectomy	1 m	3 m	6 m	12 m
Timepoint	-t_1_	0	t_1_	t_2_	t_3_	t_4_
Enrolment:						
Eligibility screen	X					
Informed consent	X					
Intervention: RARC or ORC		X				
Assessments:						
Quality of Life	X		X	X	X	X
Patient based healthcare usage	X	Monthly				
Clinical data			X	X		X

### Sample size

Based on an overall complication percentage of 65% in the ORC [3] a sample size of 338 (2 × 169) patients is required (power 80%; alpha 5%) to detect a decrease in the overall complication rate of 15%, i.e. from 65 to 50%.

### Recruitment

Urologists from 23 Dutch centres are involved in patient recruitment. Patient information has been designed in close cooperation with the patient society, i.e. representatives of the Bladder Cancer Patient Society “Leven met Blaas- of Nierkanker” are full members of the RACE project team. Both the support of the Dutch Urology Association and Bladder Cancer Patient Society “Leven met Blaas- of Nierkanker” will help us to include sufficient patients in the trial. Screening and recruitment will continue until the target population (*n* = 338) is achieved.

### Data collection methods and data management

Questionnaires will be administered web-based or paper-based, processed and stored digitally using a validated data management system (eCRF, Research Manager). Participant application forms (provided by urologists in participating centres), medical history, and clinical data will be registered, processed and stored using eCRF Research Manager. Data will be handled according to the Dutch law (Dutch Data Protection Act). Data will be anonymized by a unique identification number.

### Statistical methods

#### Outcomes

Effects on complications, disease free survival, blood transfusion and organ injury will be calculated as rate differences with 95% confidence intervals. Effects of surgery on quality of life, pain after surgery, time to return to full activity, operating time and hospital stay will be calculated as mean differences with 95% confidence intervals. All analyses will be performed on an intention-to-treat basis. To account for potential confounders (including confounding by indication), we will use both multivariate regression models and propensity score methods. Furthermore, a rule-out approach will be used for sensitivity analysis [26] to illustrate how strongly a single unmeasured binary confounder would have to be associated with both the intervention and the end point to fully explain eventual significant findings. Multiple imputation using 10 imputed data sets will be used to handle missing data.

#### Cost effectiveness analysis

The economic evaluation will be conducted conform current guidelines [27]. Primary outcome measures for the economic evaluation are societal costs, complications, and quality-adjusted life years (QALYs). To obtain QALYs, the derived health states from the EQ-5D-5 L are converted into utility scores. From these utility scores QALYs will be calculated for each patient using the Area Under the Curve (AUC) method. Resource use will be measured prospectively on a patient level using medical records and patient-completed questionnaires. The friction cost-method will be applied following the Dutch guidelines [27]. Resource use will be multiplied with the corresponding unit costs to obtain total costs for each participating patient. The Dutch guidelines for costing research will be used [27]. For units of care/resources where no guideline or standard price is available actual costs will be determined. Costs of the interventions, RARC and ORC, will be determined through activity-based costing. For RARC, the cost per procedure will depend on the anticipated throughput of patients per year. We will therefore explore a number of scenarios with different throughput.

An incremental cost-effectiveness ratio (ICER), i.e. cost per QALY gained, will be computed. Uncertainty will be determined using the bootstrap method. A cost-effectiveness acceptability curve will be derived to visualize the probability of RARC and ORC being cost-effective, over different thresholds. In addition, the impact of uncertainty surrounding important parameters (for example unit costs) on the ICER will be explored using one-way sensitivity analyses.

While the main purpose of this study involves the comparison between RARC and ORC, a third approach is laparoscopic-assisted cystectomy (LRC). Although this approach is performed in only one Dutch medical centre (i.e. Maxima Medical Centre in Veldhoven), it is interesting to additionally explore the added value of RARC and ORC compared to LRC. During the RACE study, the Maxima Medical Centre will collect the data as described above in the patients that are treated with LRC. Because this involves a small number of patients, decision analytic modelling will be performed to synthesize data from the main RACE study with this additional sample. This will allow us to explore the cost-effectiveness of the three techniques. The model will be constructed in close collaboration with clinical experts, and will comply with the latest guidelines on decision-analytic modelling in health care [28].

### Data monitoring

Participation in the RACE-study carries no risks additional to those associated with standard care. Therefore, no Data Monitoring Committee (DMC) is needed. However, the study is conducted in accordance with principles of Good Clinical Practice. For this reason, we have developed a monitoring plan for data collection, aiming for:

1. Full monitoring of informed consents;
2. Monitoring of the first three patients per center. In case of no violation ad random 10% of patients, to ensure data quality.
3. Source data verification in 10% of the included patients.

## Ethics and dissemination

### Research ethics approval and amendments

The study protocol, amendments, informed consent form and patient information brochure have been reviewed and approved by the sponsor (ZonMw, The Netherlands Organization for Health Research and Development) and the accredited medical ethics committee (Commission for Research in Human Subjects, in Dutch: Commissie Mensgebonden Onderzoek (CMO), region Arnhem – Nijmegen, the Netherlands). Since the enforcement of the Centrale Commissie Mensgebonden Onderzoek (CCMO) External Review Directive in 2012, local medical ethical committees of local participating centers are no longer involved in reviewing the study protocol of multicenter research in the Netherlands and ethical approval obtained, covers all participating centers.

### Consent or assent

Potential participants will be informed by the urologist. Informed consent documents of all participants are obtained and co-signed by clinician who provided the patient information.

### Dissemination policy

Results of the RACE study will be communicated to participants, healthcare professionals and the public through newsletters, publications in peer-reviewed journals and presentations on (inter)national meetings of the Dutch Urology Association and patient society.

## Discussion

Radical cystectomy with pelvic lymphadenectomy and urinary diversion is the standard of care for both high-risk non-muscle-invasive bladder cancer and muscle-invasive disease [1]. Due to improved anaesthesia, better surgical techniques, and centralization of care in high volume centers, reduction in morbidity and mortality has occurred in recent years. Furthermore, the ERAS protocol has also helped to reduce the length of stay and complications [29]. RARC has been suggested to potentially improve the perioperative morbidity. However, high-level evidence of any benefit from RARC over ORC of methodologically sound clinical studies is lacking. This may reflect the complex nature of this procedure as well as the complex decision-making process of clinicians selecting therapies, their experience with the technique under study, and on the decisions and actions of patients, including whether they accept or prefer a certain surgical technique. The impact of these issues on effectiveness may be difficult or impractical to assess in a traditional RCT, in which treatment assignments are randomized and may therefore lead to a bias due to the expertise of the surgeons in a center. So far, four prospective trials have compared RARC with ORC, but each of them has been limited by sample size, design, or their application of RARC with extra-corporeal reconstruction [11, 13, 14, 30].

A comparative effectiveness trial aims to provide high-quality evidence to help patients and clinicians make informed clinical decisions and to assist health systems in improving the quality and cost-effectiveness of clinical care. The major strength of our design is that we compare the preferred treatment strategy (RARC versus ORC) of several centers. Not only the surgeon, but also the team and caseload will influence the outcome for patients. With this comparative effectiveness study we compare outcomes of standardized procedures performed by surgical teams of 23 hospitals (12 ORC, 10 RARC and 1 LRC). The results will provide a valid estimate of the real effectiveness in daily clinical practice. Furthermore, we will be able to study whether the distribution of robotic systems over hospitals and patient allocation to these hospitals is indeed more or less random. Nevertheless, we will also perform propensity score analyses to adjustment for potential confounding (by indication).

Another strength of our study is that only centers that already performed at least 40 RARC-cases and have a caseload of at least 20 cases per year can participate in this study. Several studies have described the learning curve of RARC, which is estimated to be between 16 and 30 cases [17, 19]. This implies that in our study all centers have passed the learning curve.

At the time of submission of this manuscript, 273 participants have been enrolled in the study. Patient recruitment will continue until September 2018 to achieve the calculated sample size of 338 participants. Due to our experiences and the accrual rate so far, we are confident that we will be able to meet the target sample size and to quantify the effects of RARC as compared to ORC. With the results of this study we aim to contribute to the development of evidence-based guidelines regarding which of the treatment options (RARC or ORC) is the most (cost-)effective. When RARC appears to be cost-effective it might become the standard of care for the studied patient population. Patients who met the exclusion criteria (prior extensive abdominal surgery or inability for steep Trendelenburg position) should then be redirected to experienced centers for open surgery. We expect that about 75% of the cystectomies can be performed with robotic assistance. For RARC to become cost-effective and taking into account a minimal caseload of 45 cases per year due to the learning curve, centralization seems a prerequisite. We will therefore take this centralization question into account in our final analytical decision model and budget impact analysis. If RARC is not cost-effective, ORC should remain the standard treatment, which should be advocated through both international papers, updated guidelines, and various websites presenting our results. For ORC we will also model which centralization option appears to be most promising.

Moreover, we are currently collaborating with researchers from both the RAZOR and iROC trial to enable future data-sharing to perform an individual patient data meta-analysis as to identify relevant subgroups that benefit more or less and to improve the power of studying complication rates and survival.

### Trial status

Recruitment started in March 2016 and is currently ongoing. A total of 273 patients were enrolled by mid- March 2018 and we expect to finish inclusion by the end of 2018. One year followup of last patient and statistical analyses expected by the end of 2019.

## Abbreviations

BCI: Bladder Cancer Index
CCMO: Centrale Commissie Mensgebonden Onderzoek
CEA: Cost-Effectiveness Analysis
CMO: Commissie Mensgebonden Onderzoek
EQ-5D-5 L: EuroQol-5 Dimensions-5 Levels
FACT-Bl-Cys: Functional Assessment of Cancer Therapy–Bladder cancer Cystectomy
ICER: Incremental cost-effectiveness ratio
iMCQ: Medical Consumption Questionnaire
iPCQ: Productivity Cost Questionnaire
iROC: Trial to compare robotically assisted radical cystectomy with open radical cystectomy
LRC: Laparoscopic-assisted cystectomy
MREC: Medical Research Ethics Committee
NFU: Netherlands Federation of University Medical Centres
NTR: Nederlands Trial Register (Dutch Trial Registry)
ORC: Open radical cystectomy
QALY: Quality-adjusted Life Years
RACE: Radical Cystectomy Evaluation
RARC: Robot-assisted radical cystectomy
RAZOR: RAndomiZed Open vs Robotic cystectomy
VAS: Visual analogue scale
WMO: Dutch Medical Research Involving Human Subjects Act
